# CA153 in Breast Secretions as a Potential Molecular Marker for Diagnosing Breast Cancer: A Meta Analysis

**DOI:** 10.1371/journal.pone.0163030

**Published:** 2016-09-16

**Authors:** Shifu Tang, Lili Wei, Yifan Sun, Fang Zhou, Shengbo Zhu, Renqi Yang, Yiyong Huang, Hongyu Zhang, Hong Xu, Jianqing Yang

**Affiliations:** 1 Department of Laboratory Medicine, the Third Affiliated Hospital of Guangxi University of Chinese Medicine, Guangxi, China; 2 Department of Internal Medicine, the Third Affiliated Hospital of Guangxi University of Chinese Medicine, Guangxi, China; 3 Department of Rheumatology, the Fourth Affiliated Hospital of Guangxi Medical University, Guangxi, China; 4 Department of General Surgery, the Third Affiliated Hospital of Guangxi University of Chinese Medicine, Guangxi, China; University of South Alabama Mitchell Cancer Institute, UNITED STATES

## Abstract

**Purpose:**

Many studies have reported that carbohydrate antigen 153 (CA153) in breast secretions (BS) can discriminate breast cancer (BC) patients from healthy individuals, indicating CA153 in BS as a potential index for BC. This meta-analysis aimed to evaluate the actual diagnostic value of CA153 in BS.

**Methods:**

Related papers were obtained from Pubmed, Embase, Scopus, Ovid, Sciverse, the Cochrane library, Chinese Biomedical literature Database (CBM), Technology of Chongqing (VIP), Wan Fang Data, and Chinese National Knowledge Infrastructure (CNKI). Pooled sensitivity, specificity, and diagnostic odds ratio (DOR) of CA153 in BS for BC diagnosis were analyzed with the random effect model. SROC and the area under the curve (AUC) were applied to assess overall diagnostic efficiency.

**Results:**

This meta-analysis included five studies with a total of 329 BC patients and 381 healthy subjects. For CA153 in BS, the summary sensitivity, specificity, and DOR to diagnose BC were 0.63 (95% confidence interval (CI): 0.57∼0.68), 0.82 (95% CI: 0.78∼0.86), and 9.18 (95% CI: 4.22∼19.95), respectively. Furthermore, the AUC of BS CA153 in the diagnosis of BC was 0.8614.

**Conclusions:**

CA153 in BS is a valuable molecular marker in diagnosing BC and should be applied in standard clinical practices of BC screening upon confirmation of our findings in a larger prospective study.

## Introduction

BC is now the most frequently diagnosed female cancer around the world [1, 2]. Due to treatment advances, the 5-year survival rate of BC patients has being increased in the past decades [3]. However, if BC is diagnosed in late stage, the treatment is rather troublesome and prognosis is very bad [4]. Therefore, enormous efforts has been exerted on the development of diagnostic tools for early-stage BC. Until now, many BC screening methods have been invented to detect early BC. Breast self-examination, mammography, ultrasound, galactography, and exfoliative cytology are the commonly used techniques to screen early-stage BC [1]. Although these techniques have apparently improved the detection rate of early BC, there are some limitations of the above methods. For example, the early diagnosis benefit of mammography remains controversial in women younger than 50 years [5]; the false positive rate of mammography and ultrasound is too high to be tolerated [6]; breast self-examination cannot find impalpable BC [7]; galactography and exfoliative have low sensitivity in detecting BC [8, 9]. Hence, new methods are necessary for enhancing the screening efficiency of early BC.

Many studies have shown that BS could facilitate the diagnosis of early BC. BS, being secreted from the ductal and lobular epithelium of breast, contains higher concentrated proteins than serum and is only located in the ductal lumen in normal pathological conditions [10]. Under conditions of BC, excessive BS from the ductal lumen can be presented in various forms such as nipple discharge, effusions, and washout fluid [7]. Excessive BS is a common complaint of breast patients and reflects the breast microenvironment [11]. Compared with normal pathological conditions, specific components accumulate in the basement membrane-bound space of intraductal carcinoma until the continuity of the basement membrane is lost during stromal invasion [10]. Thus, many BS-based indexes were developed to improve the diagnosis of BC, especially early BC.

It has been reported that CA153 in BS has significant diagnostic value for BC. CA153, a transmembrane glycoprotein, was found as the first breast cancer-associated antigen in 1984 [12]. In 1996, Pinto MM etc. reported the levels of CA153 in BS from BC were higher than that from healthy controls [13]. Subsequently, it was first reported that CA153 in BS can differentiate malignant breast cancers from benign breast diseases and is a more valuable biomarker for diagnosing BC compared with mammography [14]. Later,many researchers also investigated the diagnostic role of CA153 in BS [10, 15–17].

To further confirm whether CA153 in BS can be used as a reliable diagnostic biomarker for BC, particularly early BC, we performed a meta-analysis by pooling related published studies. Our study results verified CA153 in BS has a moderate diagnostic value for BC and should be included into routine screening practices, on condition that our results are testified in a large-scale population study.

## Methods

### Literature retrieving strategy

Relevant papers were obtained by searching the "Title and Abstract" field in the following databases: Pubmed, Embase, Scopus, Ovid, Sciverse, the Cochrane library, CBM, VIP, Wan Fang Data, and CNKI up to 10 May, 2016. The keywords for searching literature were: (1) mammary gland* or breast*; (2) cancer* or tumo* or carcinoma* or neoplasm*; (3) nipple* or breast duct*; (4) discharge* or secretion* or fluid* or effusion*; (5) cancer antigen 153 or carbohydrate antigen 153 or cancer associated antigen 153 or CA153. The search was not restricted by publication time or status except for human studies written in English. Furthermore, the references of included articles and related articles were manually skimmed to find out potential studies.

### Eligible criteria for the included studies

Any related articles were carefully evaluated by two researchers (SFT and LLW) independently on the basis of titles and abstracts to screen potential qualified studies for further reading their full texts. Disagreement was resolved by full discussion to come consensus. If key data is deficient, We wrote to the authors to acquire the data. Every included study have to confirm to the criteria: (1) The diagnosis of BC was based on histopathological examination; (2) Corresponding control individuals were identified not to have BC or a history of any type of cancer; (3) Any sample from nipples were obtained before any treatment; (4) The CEA levels were determined in breast secretions; (5) The studies had values of sensitivity and specificity (or there was the possibility of deriving those values from the articles), and a specific cut-off value; (6) Only studies with more than 25 cases and matched controls were included. Any study was not included if it belonged to: (1) Review, case report, and meeting abstract; (2) repeat publications; (3) ineligible patients and controls; (4) a diagnosis not based on biopsy and/or absence of a definite cut-off value.

### Data extraction

Eligible studies were reviewed by two researchers (SFT and YFS) respectively. The extracted data are: the first author, publication time, country, journal, sample size, features of patients and controls, index measuring methods, the cut-off value, and the data in a four-fold table. Any disagreement was solved by a full debate of all authors until a consensus was achieved.

### Quality assessment

The quality of all studies was assessed independently by two investigators (SFT and FZ) with QUADAS-2 (Quality Assessment of Diagnostic Accuracy Studies 2). QUADAS-2 is a famous tool featured with four domains (patient selection, index test, reference standard, and flow and timing) that facilitate the measurement of bias risk and concerns about applicability (rating these parameters as "high", "low", and "unclear") [18].

### Statistical analysis

The STATA 12.0 and Meta-Disc statistical software were employed to analyze the data from the eligible papers [19]. The summary indexes were obtained from the raw data of each study. The pooled indexes were presented as forest plots. Furthermore, the studies’ heterogeneity from the threshold effect was examined with the Spearman correlation analysis method. The non-threshold effect was measured with the Cochran-Q method and the test of inconsistency index (I^2^). A *P* value (≤ 0.05) and a I^2^ value (≥50)suggest the Non-threshold effect lead to the heterogeneity between these studies. If there is non-threshold effect, meta-regression was used to investigate the sources. As for publication bias, we analyzed the included studies with Begg's test and Egger's test. A low P value (≤ 0.05) was considered statistically significant.

## Results

### Literature selection

**Fig 1:**
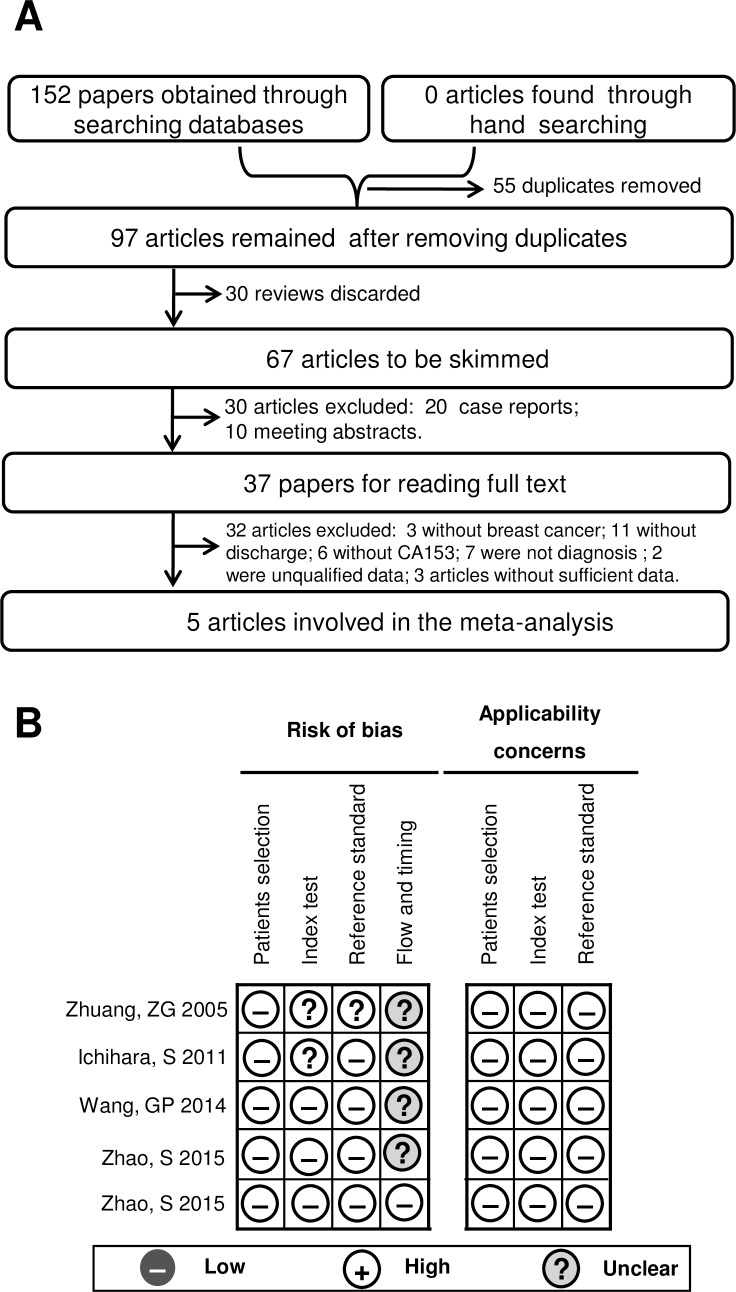
A literature screening flow diagram and quality assessment schematic diagram for the included articles. **(A)** A flow diagram of screening eligible studies. **(B)** Presentation of data quality evaluated with the QUADAS-2 tool, showing “risk of bias” and “concerns of applicability” of each eligible study (with risk of bias in the “flow and timing” domain).

One hundred fifty-two papers were obtained by applying our literature searching strategy (database searching and hand retrieval). As displayed in Fig 1A, The screening process picked out 5 high-quality articles that confirmed to our inclusion criteria. Two of the authors were asked for experimental details of their study subjects. The authors responded.

### Key study features

All eligible studies were published from 2005 to 2015 and contained 329 BC patients and 381 controls. All of our study characteristics are listed in S1 Table.

### Quality evaluation

The qualified studies were assessed using the QUADAS-2. As seen in Fig 1B, the five eligible studies were of high quality in terms of most indicators, but a major bias was verified. The major bias was from the “flow and timing” domain since the definite interval and interventions between nipple discharge collection and operation treatment were not stated in most of these studies.

### Heterogeneity analysis

**Fig 2:**
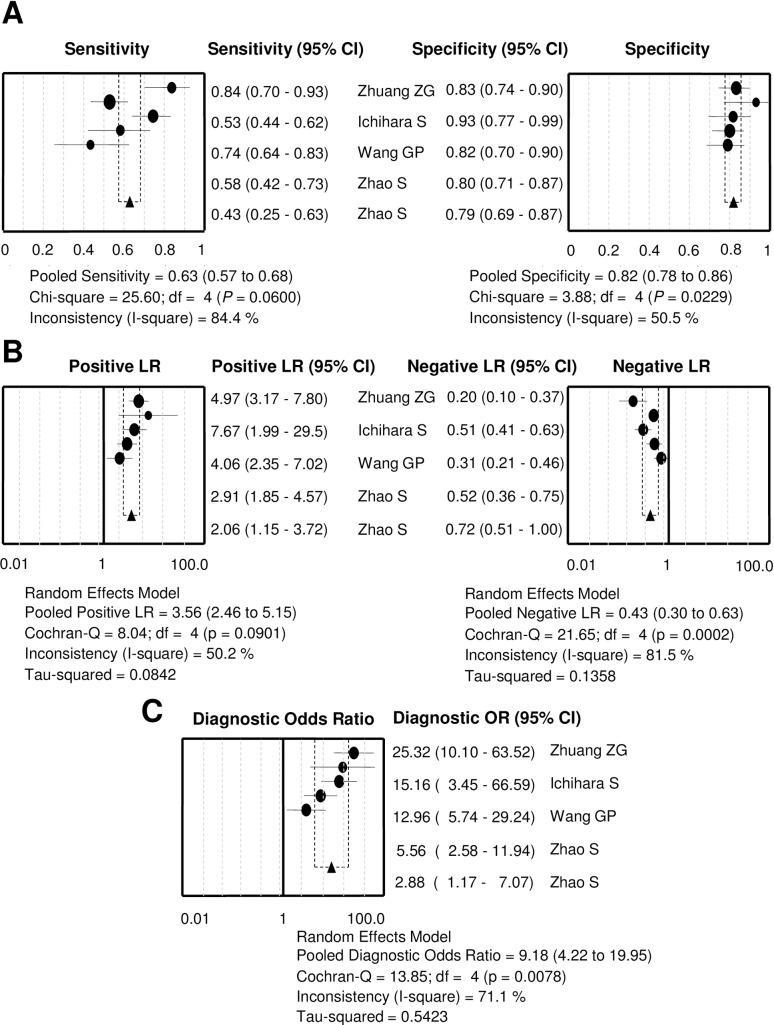
The summary diagnostic indices of CA153 in BS for BC diagnosis exhibited in forest plots. **(A)** sensitivity and specificity; **(B)** positive LR and negative LR; **(C)** DOR. These pooled indices indicate that CA153 in BS could be a useful indicator for the noninvasive diagnosis of BC. The individual index for each study is represented by circles, and the combined indices are shown as triangles.

Heterogeneity analysis plays an essential role in exploring the factors that might interfere with the accuracy of diagnostic indices and in evaluating the pooling appropriateness of statistical indices of primary studies [18]. To verify whether there is heterogeneity of CA153 in our eligible studies, we firstly calculated the correlation coefficient and *P* value between the logit of sensitivity and the logit of 1-specificity by using the Spearman test to detect the threshold effect. The Spearman correlation coefficient and *P* value were -0.400 and 0.505 (>0.05) respectively, showing that heterogeneity of CA153 caused by the threshold effect did not exist in the five studies. Because heterogeneity can derive from the non-threshold effect, we employed the inconsistency index (I^2^) to investigate the heterogeneity caused by the non-threshold effect. The value of I^2^ of all diagnostic indexes was greater than 50% (as seen in Fig 2), implying that there is heterogeneity resulted from the non-threshold effect in these studies.

### Data analysis

**Fig 3:**
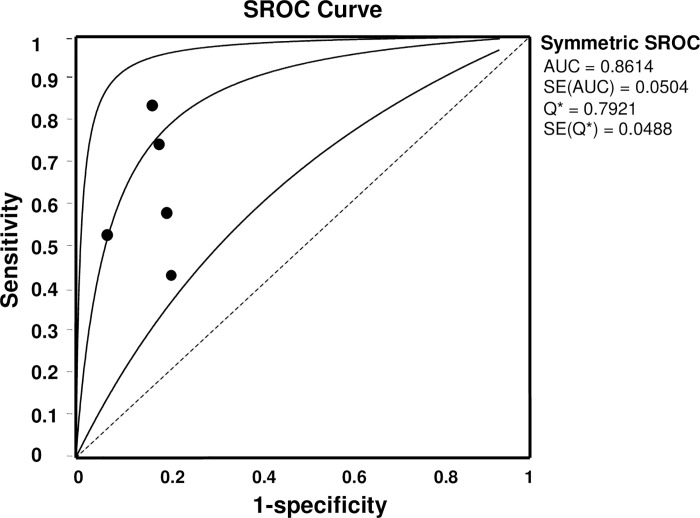
The overall diagnostic performance of CA153 in BS, shown by SROC. Each circle represents a study. The SROC curve is symmetric and the AUC is 0.8614, suggesting a moderate diagnostic accuracy for BC.

Because only the non-threshold effect may lead to potential heterogeneity, the random effect model was used to assess the overall diagnostic performance of CA153 in BS for BC. For CA153, the summary indexes of these studies were presented in forest plots (Fig 2). The pooled sensitivity and specificity of CA153 were 0.63 (95% CI: 0.57∼0.68) and 0.82 (95% CI: 0.78∼0.86) respectively in diagnosing BC (Fig 2A). The pooled PLR and NLR were 3.56 (95% CI: 2.46∼5.15) and 0.43 (95% CI: 0.30∼0.63) respectively (Fig 2B). The total DOR (Fig 2C) and the area under SROC (Fig 3) were 9.18 (95% CI: 4.22∼19.95) and 86.14% respectively, indicating a moderate accuracy of nipple secretion CA153 in diagnosing BC.

### Meta-regression analysis

As shown in Fig 2, the heterogeneity of these studies was caused by the non-threshold effect. To find the underlying causes, The meta-regression was used to reveal the influence of various study characteristics such as age, sample size, and histopathological type. Unexpectedly, no clues belonged to specific causal factors.

### Publication bias

**Fig 4:**
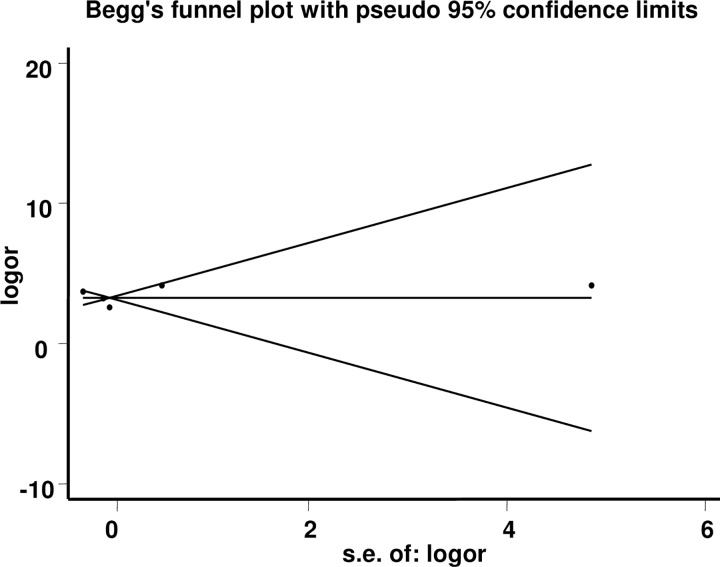
Publication bias is exhibited with a funnel plot. Each point represents one study, and results demonstrate that publication bias does not exist.

Publication bias exerts important effect on the accuracy of pooled diagnostic indexes. To investigate publication bias in this study, the Begg’s funnel plot and Egger’s test were used. Results demonstrated that there is not publication bias in this meta-analysis. The Egger’s test displays funnel plot symmetry (P > 0.05), and as shown in Fig 4, the Begg’s funnel plot shows that this meta-analysis has no publication bias (*P* Egger's test = 0.805, 95% CI = –4.11∼4.69).

## Discussion

CA153 is a more sensitive and specific biomarker for BC than CEA(cancer embryonic antigen) [20]. Serum CA153 is not a useful diagnostic biomarker for BC, especially early BC [14, 21], but CA153 in BS has been shown by many labs to be of significant value in diagnosing BC. To our knowledge, this study is the first systematic review of CA153 in BS as a diagnostic biomarker for BC since its potential was first found by Hilkens in 1984 [20]. This meta-analysis confirmed that CA153 in BS could be a valuable diagnostic index for BC and it had an AUC of 0.8614, with overall 63% (95% CI: 0.57∼0.68) sensitivity and 0.82 (95% CI: 0.78∼0.86) specificity, displaying its capability as a noninvasive screening biomarker for BC. AUC measures the total diagnostic performance, and the ideal value is infinitely approximate to 1 [22]. An AUC of 0.75∼0.92 is considered to be good according to the accuracy evaluation standard [22, 23]. In this study, an AUC value of 0.8614 strongly supported that CA153 in BS has a moderate diagnostic value for BC.

Meta-analysis is invariable accompanied by different degrees of heterogeneity [19]. In this meta-analysis, we first showed that there is no heterogeneity caused by the threshold effect. But, heterogeneity from the non-threshold effect exists. Then we analyzed the underlying sources of heterogeneity with the meta-regression method but found no significant factors, which may result from the relative small number of articles. Publication bias can also seriously distort conclusions of a meta-analysis if only studies with positive conclusions are obtained for a pooled analysis [24]. Luckily, publication bias does not exist in our meta-analysis in spite of a small number of papers.

CA153 in BS has many advantages as a diagnostic biomarker of BC. First of all, CA153 in BS could be a promising biomarker for the diagnosis of early BC. Although relevant studies are so few that the diagnostic role of BS CA153 for BC cannot supported by a meta-analysis, two studies demonstrated CA153 in BS can discriminate early BC from healthy controls, indicating its diagnostic value for early-stage BC [10, 14]. Second, the detection of CA153 in BS is safe and accessible. Mammography has been the leading approach for BC screening [25]. But, the achilles’ heel of this technique is the risk of radiation-caused BC during mammography screening [26]. In contrast, measurement of CA153 in BS is noninvasive and safe expect for ductal lavage and fine needle aspiration [7]. Moreover, the relative low price of CA153 testing make it accessible to the public. As for mammography or MRI, the high price of these detection tools often makes patients unaffordable because of the need of expensive specific instruments, skillful technicians, and spacious working area [27, 28]. Thirdly, detection of CA153 in BS may be a useful auxiliary way in diagnosing BC. As the standard screening approach, mammography cannot find 10%∼40% BC [29]. Any serum indicator has not been found to aid in diagnosing BC [30]. Because of high concentrations in BS, the combination of mammography and detection of CA153 in BS should increase the positive rate of BC screening. Lastly, the levels of CA153 in breast secretions may be closely correlated with the stage of breast cancer and any treatment. In theory, the higher the stage of breast cancer, the higher the CA153 concentration in breast secretions; the levels of CA153 in breast secretions would be significantly decreased after accepting effective treatments. One of the 5 primary studies showed an opposite result that the levels of CA153 in breast secretions from invasive breast cancer are lower than that from DCIS [10], which might result from the blockage of breast ducts during the breast cancer expansion. However, whether the levels of CA153 in breast secretions respond to any clinical treatment has not been reported. This may be caused by the lack of breast secretions after clinical treatment. In a word, CA153 in BS is a good index suitable for screening BC and deserves exploring its diagnostic role for early-stage BC.

As every coin has two sides, there are some defects about this meta-analysis. Above all, the total sample size in this study is relative small, which could impose potential effect on our conclusions. Thus, more large studies are needed to confirm our findings. Furthermore, our conclusions may not be suitable for all kinds of BC subtypes. It is well known that heterogeneity is a prominent feature of BC [31]. This meta-analysis is on the basis of studies that did not identify the subtypes of BC because we did not find any literature suggesting specific sub-type of breast cancer while writing this meta-analysis. Moreover, the pooled sensitivity of 63% is low in this meta-analysis, which may be caused by the heterogeneous nature of BC [31]. Our previous study also showed that CEA in BS has a low sensitivity of 58% [95% CI: 52%∼63%] in diagnosing BC [32]. Parallel testing of CA153 and CEA in BS will improve the diagnostic efficiency of BC. Finally, there are some biases in this meta-analysis. The risk factors such as smoking and alcohol consumption might increase the levels of CA153 in breast secretions to lead false positive results. It is well-known that smoking, alcohol consumption or obesity increases the risk of breast cancer [33–36]. Moreover, alcohol can promote breast epithelial proliferation and breast cancer cell invasion [37, 38], indicating alcohol can promote breast cancer development. Although there is no evidence demonstrating that smoking or alcohol consumption increases the concentration of CA153 in breast secretions from healthy subjects, this may exist and lead to false positive results when compared with the cut-off value of CA153 in breast secretions calculated from the control group without smoking and alcohol consumption. Therefore, when encountering positive results in screening breast cancer with the test of CA153 in breast secretions, it is advisable that employment of other tools in series including mammography and clinical breast examination could effectively reduce the false positive results. In the long run, it is better to establish a specific cut-off value of CA153 in breast secretions for the population with smoking or alcohol consumption. In the "flow and timing" domain, only one of the five included studies described the design of "flow and timing." In the experimental process, only three of these studies were done by double blind.

In short, our results has demonstrated that detection of CA153 in BS is of valuable diagnostic role for BC. If verified in a larger prospective study, CA153 in BS could be used to screen BC in the future.

## Supporting Information

S1 PRISMA Checklist (DOC)

S1 Table Key characteristics of the studies included in this meta-analysis. (DOC)

## References

[1] Chen W, Zheng R, Baade PD, Zhang S, Zeng H, Bray F, et al(2015). Cancer statistics in China, 2015. CA Cancer J Clin 66: 115–32. 10.3322/caac.21338 26808342

[2] Fan L, Strasser-Weippl K, Li JJ, St Louis J, Finkelstein DM, Yu KD, et al(2014). Breast cancer in China. Lancet Oncol 15: e279–89. 10.1016/S1470-2045(13)70567-9 24872111

[3] Gewefel H, Salhia B(2014). Breast cancer in adolescent and young adult women. Clin Breast Cancer 14: 390–5. 10.1016/j.clbc.2014.06.002 25034440

[4] Ferlay J, Shin HR, Bray F, Forman D, Mathers C, Parkin DM(2010). Estimates of worldwide burden of cancer in 2008: GLOBOCAN 2008. Int J Cancer 127: 2893–917. 10.1002/ijc.25516 21351269

[5] Siu AL(2009). Screening for breast cancer: U.S. Preventive Services Task Force recommendation statement. Ann Intern Med 151: 716–26, W-236. 10.7326/0003-4819-151-10-200911170-00008 19920272

[6] Yip CH, Cazap E, Anderson BO, Bright KL, Caleffi M, Cardoso F, et al(2011). Breast cancer management in middle-resource countries (MRCs): consensus statement from the Breast Health Global Initiative. Breast 20 Suppl 2: S12–9. 10.1016/j.breast.2011.02.015 21388811

[7] Mannello F(2008). Analysis of the intraductal microenvironment for the early diagnosis of breast cancer: identification of biomarkers in nipple-aspirate fluids. Expert Opin Med Diagn 2: 1221–31. 10.1517/17530059.2.11.1221 23496682

[8] Dinkel HP, Trusen A, Gassel AM, Rominger M, Lourens S, Muller T, et al(2000). Predictive value of galactographic patterns for benign and malignant neoplasms of the breast in patients with nipple discharge. Br J Radiol 73: 706–14. 11089460 10.1259/bjr.73.871.11089460

[9] Sauter ER, Ehya H, Babb J, Diamandis E, Daly M, Klein-Szanto A, et al(1999). Biological markers of risk in nipple aspirate fluid are associated with residual cancer and tumour size. Br J Cancer 81: 1222–7. 10584885 10.1038/sj.bjc.6690832 PMC2374332

[10] Ichihara S, Moritani S, Hasegawa M, Shiraiwa M, Oiwa M, Endo T, et al(2011). Measurement of human epidermal growth factor receptor type-2 extracellular domain and cancer antigen 15–3 levels in needle washout fluid: A potential adjunct to the cytological diagnosis of breast cancer. Virchows Archiv 458: 547–559. 10.1007/s00428-011-1065-2 21437720

[11] Vargas HI, Romero L, Chlebowski RT(2002). Management of bloody nipple discharge. Curr Treat Options Oncol 3: 157–61. 12057078 10.1007/s11864-002-0061-9

[12] Jardines L(1996). Management of nipple discharge. Am Surg 62: 119–22. 8554189

[13] Pinto MM(1996). CA-15.3 assay in effusions: comparison with carcinoembryonic antigen and CA-125 assay and cytologic diagnosis. Acta Cytol 40: 437–42. 8669175 10.1159/000333895

[14] zhuang z, Yu j, Jiang p, al e(2005). the significance for expression of CA153 in nipple discharge and serum of the breast cancer. Chinese Journal of Clinical Oncology 32: 690–691.

[15] Wang G, Qin Y, Zhang J, Zhao J, Liang Y, Zhang Z, et al(2014). Nipple discharge of CA15-3, CA125, CEA and TSGF as a new biomarker panel for breast cancer. Int J Mol Sci 15: 9546–65. 10.3390/ijms15069546 24879526 PMC4100109

[16] Zhao S, Mei Y, Wang Y, Zhu J, Zheng G, Ma R(2015). Levels of CEA, CA153, CA199, CA724 and AFP in nipple discharge of breast cancer patients. Int J Clin Exp Med 8: 20837–44. 26885008 PMC4723853

[17] Zhao S, Gai X, Wang Y, Liang W, Gao H, Zhang K, et al(2015). Diagnostic Values of Carcinoembryonic Antigen, Cancer Antigen 15–3 and Cancer Antigen 125 Levels in Nipple Discharge. Chin J Physiol 58: 385–92. 10.4077/CJP.2015.BAD336 26717917

[18] Whiting PF, Rutjes AW, Westwood ME, Mallett S, Deeks JJ, Reitsma JB, et al(2011). QUADAS-2: a revised tool for the quality assessment of diagnostic accuracy studies. Ann Intern Med 155: 529–36. 10.7326/0003-4819-155-8-201110180-00009 22007046

[19] Zamora J, Abraira V, Muriel A, Khan K, Coomarasamy A(2006). Meta-DiSc: a software for meta-analysis of test accuracy data. BMC Med Res Methodol 6: 31 16836745 10.1186/1471-2288-6-31 PMC1552081

[20] Gion M, Mione R, Nascimben O, Valsecchi M, Gatti C, Leon A, et al(1991). The tumour associated antigen CA15.3 in primary breast cancer. Evaluation of 667 cases. Br J Cancer 63: 809–13. 2039707 10.1038/bjc.1991.179 PMC1972400

[21] Kurebayashi (2004). Biomarkers in breast cancer. Gan To Kagaku Ryoho 31: 1021–6. 15272579

[22] Jones CM, Athanasiou T(2005). Summary receiver operating characteristic curve analysis techniques in the evaluation of diagnostic tests. Ann Thorac Surg 79: 16–20. 15620907 10.1016/j.athoracsur.2004.09.040

[23] Walter SD(2002). Properties of the summary receiver operating characteristic (SROC) curve for diagnostic test data. Stat Med 21: 1237–56. 12111876 10.1002/sim.1099

[24] Begg CB, Mazumdar M(1994). Operating characteristics of a rank correlation test for publication bias. Biometrics 50: 1088–101. 7786990

[25] Mantas D, Markopoulos C(2016). Screening mammography: Usefulness beyond early detection of breast cancer. Atherosclerosis 248: 1 10.1016/j.atherosclerosis.2016.02.019 26970928

[26] Ali RM, England A, McEntee MF, Hogg P(2015). A method for calculating effective lifetime risk of radiation-induced cancer from screening mammography. An international journal of diagnostic imaging and radiation therapy 21: 298–303.

[27] Loberg M, Lousdal ML, Bretthauer M, Kalager M(2015). Benefits and harms of mammography screening. Breast Cancer Res 17: 63 10.1186/s13058-015-0525-z 25928287 PMC4415291

[28] Morrow M, Waters J, Morris E(2011). MRI for breast cancer screening, diagnosis, and treatment. Lancet 378: 1804–11. 10.1016/S0140-6736(11)61350-0 22098853

[29] Kolb TM, Lichy J, Newhouse JH(2002). Comparison of the performance of screening mammography, physical examination, and breast US and evaluation of factors that influence them: an analysis of 27,825 patient evaluations. Radiology 225: 165–75. 12355001 10.1148/radiol.2251011667

[30] Brooks(2009). Breast cancer screening and biomarkers. Methods Mol Biol 472: 307–21. 10.1007/978-1-60327-492-0_13 19107439

[31] Network CGA(2012). Comprehensive molecular portraits of human breast tumours. Nature 490: 61–70. 10.1038/nature11412 23000897 PMC3465532

[32] Tang S, Zhou F, Sun Y, Wei L, Zhu S, Yang R, et al(2016). CEA in breast ductal secretions as a promising biomarker for the diagnosis of breast cancer: a systematic review and meta-analysis. Breast Cancer10.1007/s12282-016-0680-9 26898373

[33] Shield KD, Soerjomataram I, Rehm J(2016). Alcohol Use and Breast Cancer: A Critical Review. Alcohol Clin Exp Res 40: 1166–81. 10.1111/acer.13071 27130687

[34] Chen WY, Rosner B, Hankinson SE, Colditz GA, Willett WC(2011). Moderate alcohol consumption during adult life, drinking patterns, and breast cancer risk. Jama 306: 1884–90. 10.1001/jama.2011.1590 22045766 PMC3292347

[35] Macacu A, Autier P, Boniol M, Boyle P(2015). Active and passive smoking and risk of breast cancer: a meta-analysis. Breast Cancer Res Treat 154: 213–24. 10.1007/s10549-015-3628-4 26546245

[36] Howe LR, Subbaramaiah K, Hudis CA, Dannenberg AJ(2013). Molecular pathways: adipose inflammation as a mediator of obesity-associated cancer. Clin Cancer Res 19: 6074–83. 10.1158/1078-0432.CCR-12-2603 23958744 PMC3891839

[37] Schennink A, Trott JF, Berryhill GE, Donovan CE, Manjarin R, VanKlompenberg MK, et al(2015). Alcohol intake stimulates epithelial proliferation in an authentic model of the human breast. Reprod Toxicol 54: 93–100. 10.1016/j.reprotox.2014.10.020 25450420

[38] Wong AW, Paulson QX, Hong J, Stubbins RE, Poh K, Schrader E, et al(2011). Alcohol promotes breast cancer cell invasion by regulating the Nm23-ITGA5 pathway. J Exp Clin Cancer Res 30: 75 10.1186/1756-9966-30-75 21838876 PMC3170226

