# Supplementary material for: CA153 in Breast Secretions as a Potential Molecular Marker for Diagnosing Breast Cancer: A Meta Analysis
Source: PLoS One. 2016 Sep 16;11(9):e0163030. doi: 10.1371/journal.pone.0163030 (PMC5026335; doi:10.1371/journal.pone.0163030)
Supplement: S1 Table — (DOC) [file pone.0163030.s002.doc]

S1 Table. Key characteristics of the studies included in this meta-analysis.

| First author (year) | Country | Patients/  controls | Patient or  control age (years) | Histological-  pathological types | Assay type | Cut-off values | TP | | FP | FN | TN |
| --- | --- | --- | --- | --- | --- | --- | --- | --- | --- | --- | --- |
| Zhuang ZG, (2005) | China | 49/101 | 32 to 65 years old/ 27 to 35 years old | including invasive ductal carcinoma, medullary carcinoma, mucinous carcinoma, and intraductal carcinoma. | ELISA | 40(μg/L) | 41 | | 17 | 8 | 84 |
| Ichihara S， (2011) | Japan | 121/29 | 30 to 85 years old/ 15 to 86 years old | 26 pure DCIS,81 invasive ductal carcinomas, and 14 rare types. | ELISA | 4(U/ml), the washout fluid was diluted with normal saline(1:28). | 64 | | 2 | 57 | 27 |
| Wang, GP (2014) | China | 86/60 | the mean age of patients and controls is 47.5 and 46.2 respectively. | 86 unilateral invasive ductalcarcinoma | ELISA | 35(U/ml) | 64 | | 11 | 22 | 49 |
| Zhao, S (2015) | China | 43/110 | 17 to 76 years old for all subjects. | 18 invasive ductal carcinoma；15 DCIS；10 intraductal papillary carcinoma. | ELISA | 1368.2(U/ml) | | 25 | 22 | 18 | 88 |
| Zhao, S (2015) | China | 30/81 | the mean age of patients and controls is 47.5  28 to 72 years old/20 to 64 years old | 11 invasive ductal carcinoma；  10 intraductal papillary carcinoma; 8 DCIS. | ELISA | 1368.2(U/ml) | | 13 | 17 | 17 | 64 |

TP：true positive；FP：false positive；FN：false negative；TN：true negative. DCIS:ductal carcinoma in situ；ECLIA: Electrochemiluminescence Immunoassay;
